# Exosomes in the Diagnosis of Neuropsychiatric Diseases: A Review

**DOI:** 10.3390/biology13060387

**Published:** 2024-05-28

**Authors:** Song Wu, Xinmiao Shang, Meng Guo, Lei Su, Jun Wang

**Affiliations:** 1 Autism & Depression Diagnosis and Intervention Institute, Hubei University of Technology, Wuhan 430068, China; wusong@hbut.edu.com (S.W.); 102110686@hbut.edu.cn (X.S.); 102210700@hbut.edu.cn (M.G.); 2 National “111” Center for Cellular Regulation and Molecular Pharmaceutics, Hubei Key Laboratory of Industrial Microbiology, Hubei University of Technology, Wuhan 430068, China; 3 Cooperative Innovation Center of Industrial Fermentation, Ministry of Education & Hubei Province, Hubei University of Technology, Wuhan 430068, China; 4 Shenzhen Key Laboratory of Nano-Biosensing Technology, Marshall Laboratory of Biomedical Engineering, School of Biomedical Engineering, Shenzhen University Medical School, Shenzhen University, Shenzhen 518060, China; sulei@szu.edu.cn

**Keywords:** exosome, neuropsychiatric disease, diagnosis, central nervous system disease

## Abstract

**Simple Summary:**

The diagnostic value of exosomes has been well recognized by researchers. The use of exosomal biomarkers as an adjunct diagnosis method not only improves diagnostic accuracy but can also be used for early diagnosis and disease progression differentiation, thus supporting personalized clinical treatment strategies for patients with neuropsychiatric disorders. In this paper, we summarize potential exosomal biomarkers in the diagnosis of neuropsychiatric diseases.

**Abstract:**

Exosomes are 30–150 nm small extracellular vesicles (sEVs) which are highly stable and encapsulated by a phospholipid bilayer. Exosomes contain proteins, lipids, RNAs (mRNAs, microRNAs/miRNAs, long non-coding RNAs/lncRNAs), and DNA of their parent cell. In pathological conditions, the composition of exosomes is altered, making exosomes a potential source of biomarkers for disease diagnosis. Exosomes can cross the blood–brain barrier (BBB), which is an advantage for using exosomes in the diagnosis of central nervous system (CNS) diseases. Neuropsychiatric diseases belong to the CNS diseases, and many potential diagnostic markers have been identified for neuropsychiatric diseases. Here, we review the potential diagnostic markers of exosomes in neuropsychiatric diseases and discuss the potential application of exosomal biomarkers in the early and accurate diagnosis of these diseases. Additionally, we outline the limitations and future directions of exosomes in the diagnosis of neuropsychiatric diseases.

## 1. Introduction

Extracellular vehicles (EVs) are granules that are naturally released from cells. EVs are categorized as small extracellular vesicles (sEVs, size < 200 nm) and large extracellular vesicles (lEVs, size > 200 nm) based on their size [1]. Exosomes are 30–150 nm sEVs secreted by almost all cells [2]. The process of exosome formation includes these steps: extracellular components enter the cell through a vesicle formed by membrane invagination, the vesicle exchanges materials within the cell. the membrane invaginates again to form a multivesicular body (MVB), finally, the cell releases the exosome from the MVB to the outer cytosol via an in vivo degradation pathway or a cytoplasmic fusion pathway [2,3,4]. The blood–brain barrier (BBB) is a defensive structure of endothelial cells, astrocytes and microglia, neurons, and the extracellular matrix that can prevent harmful substances entering the brain by selectively impeding the exchange of certain substances between blood and brain [5]. The unique phospholipid bilayer structure and nanoscale particle size of exosomes allow exosomes to cross the BBB [6]. Brain-derived exosomes can be isolated in peripheral body fluids, such as plasma, serum, urine, and saliva [7,8,9,10]. Exosomes can efficiently cross the BBB, and, therefore, exosomes from peripheral body fluids can be used to detect central nervous system (CNS) diseases [11,12].

Neuropsychiatric diseases, such as cognitive deficit, memory deficit, emotional deficit, volitional deficit, and behavioral deficit are deficits of the CNS caused by a variety of biological, psychological, and socio-environmental factors. Neuropsychiatric diseases are characterized by a high degree of symptom overlap with complex diagnostic procedures. Clinical diagnosis of neuropsychiatric diseases is mainly based on the assessment of the patient’s behavior, with the use of genetic, metabolic, and neuroimaging data as adjunctive diagnostic methods. Certainly, their diagnosis still needs to be improved, especially for early diagnosis, subtype differentiation, and prognostic testing. Exosomes are involved in the regulation of neuroinflammation, synaptic plasticity, the immune system, redox, and cellular communication, and are related to the pathogenesis of neuropsychiatric diseases. This review summarizes exosomal biomarkers identified from patients with neuropsychiatric diseases, including neurodevelopmental diseases, mind and will disorders, mood disorders, demyelinating diseases, and neurodegenerative diseases (Table S1). It discusses the potential of using exosomal biomarkers for the diagnosis of neuropsychiatric diseases and provides an outlook on limitations and future research directions.

## 2. Exosomes in the Diagnosis of Neurodevelopmental Diseases

**Figure 1:**
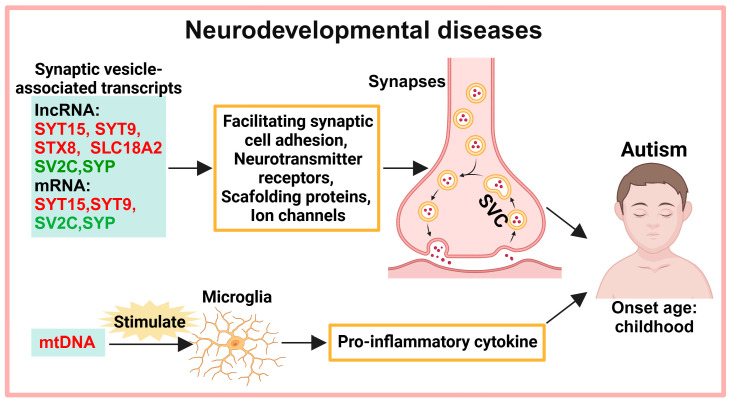
Potential exosomal diagnostic biomarkers in neurodevelopmental diseases. Abnormal expression of synaptic vesicle-associated genes and mtDNA has been detected in exosomes of patients with autism. Note: Light green boxes represent exosomal biomarkers; red font represents increased expression; green font represents decreased expression. lncRNA: long non-coding RNAs; mRNA: messenger RNA; SYT15: synaptotagmin 15; SYT9: synaptotagmin 9; STX8: syntaxin-8; SLC18A2: solute carrier protein 18 A2; SV2C: synaptic vesicle glycoprotein 2C; SYP: synaptophysin; mtDNA: mitochondrial DNA; SVC: synaptic vesicle cycling.

Neurodevelopmental diseases occur when the brain or CNS encounters barriers to growth or development. The onset of these disorders is in childhood for most patients. Neurodevelopmental diseases include autism spectrum disorder (ASD), attention deficit hyperactivity disorder (ADHD), intellectual disability (ID), etc. These diseases show a high co-occurrence rate, with overlapping symptoms, such as movement disorders, learning disabilities, and communication disorders [13]. Currently, only exosomal biomarkers for ASD have been reported in patients among these neurodevelopmental diseases (Figure 1).

### 2.1. Neurotransmitter Transmission

Neurons release neurotransmitters from synaptic vesicles to the next neuron to complete the basic function of signal transmission in the nervous system. Synaptic vesicle glycoprotein 2C (SV2C) and synaptophysin (SYP) are synaptic vesicle-associated proteins, and their lncRNAs and mRNAs are significantly reduced [14,15,16]. Synaptotagmin 15 (SYT15) and synaptotagmin 9 (SYT9) are involved in the transport and secretion of synaptic vesicles, and their lncRNAs and mRNAs are increased [14,17]. Syntaxin-8 (STX8) is a fusion protein of synaptic vesicles, and solute carrier protein 18 A2 (SLC18A2) is a vesicular monoamine transporter protein that transports amine neurotransmitters to synaptic vesicles [14,18,19], their lncRNAs are significantly increased [14].

### 2.2. MtDNA-Mediated Neuroinflammation

Mitochondrial DNA (mtDNA) increases in both serum and serum exosomes of ASD patients [20,21]. MtDNA contributes to neuroimmune dysregulation in ASD by inducing immune cells to secrete pro-inflammatory factors and activating autoimmune responses [20]. Stimulation of cultured microglia with mtDNA-enriched exosomes from the serum of ASD patients results in a significant increase in the pro-inflammatory cytokine IL-1β [21], as well as occurring in the brains of children with ASD and in mouse models of ASD [22,23,24,25]. MtDNA can enter exosomes, which has been reported by different studies [26,27,28]. One way that mtDNA enters into exosomes is as follows: pathogenic and damage-associated molecular patterns enhance the permeability of the mitochondrial outer membrane leading to leakage of mtDNA into the cytosol, and the material exchange process in early exosomes takes place in the cytosol [29].

## 3. Exosomes in the Diagnosis of Mind and Volition Disorders

**Figure 2:**
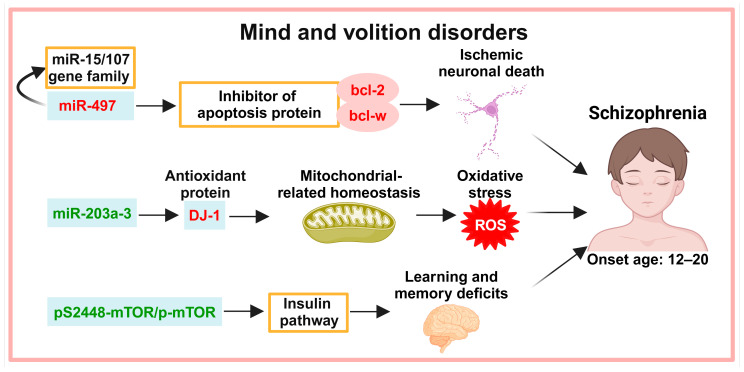
Potential exosomal diagnostic biomarkers in mind and volition disorders. Abnormal expression of miRNAs associated with apoptosis, oxidative stress, and insulin pathways was detected in patients with SCZ. Note: Light green boxes represent exosomal biomarkers; red font represents increased expression; green font represents decreased expression. miR: microRNA/miRNA; bcl-2: B-cell lymphoma 2; bcl-w: B-cell lymphoma w; DJ-1/PARK7: human protein deglycase, encoding by the PARK7 gene; ROS: reactive oxygen species; (pS2448-mTOR)/m-TOR: the ratio of the ratio of phosphorylated mammalian target of rapamycin (pS2448-mTOR) to total mammalian target of rapamycin to total mammalian target of rapamycin.

Schizophrenia (SCZ) is the most common mental disorder. The onset time of SCZ is usually from the ages of 12 to 20, and it is characterized by delusions, hallucinations, and thought and affective disorders. Exosome biomarkers in different pathways have been identified in SCZ (Figure 2).

### 3.1. Neuronal Apoptosis

miR-497 is increased in prefrontal cortex exosomes of SCZ patients [30]. miR-497 belongs to the miR-15/107 family of miRNAs, which affect cortical gene expression and are associated with neurodegenerative diseases [31], and miR-497 downregulation in cancer releases B-cell lymphoma 2 (bcl-2) and B-cell lymphoma w (bcl-w) expression, thus inhibiting apoptosis [32].

### 3.2. Oxidative Stress

Impaired antioxidant function and ROS production are potential pathogenic mechanisms in SCZ. Redox-related molecules are altered in SCZ patients [33,34]. DJ-1 is an antioxidant protein that regulates the expression of antioxidant defence genes, protecting cells from oxidative stress damage [35,36]. DJ-1 is significantly increased in serum exosomes of SCZ patients, and miR-203a-3, which targets the mRNA of DJ-1, is significantly decreased in serum exosomes of SCZ patients [37].

### 3.3. Insulin Pathways

Learning and memory deficits in SCZ are associated with insulin resistance in the brain [38]. Protein phosphorylation levels downstream of the insulin signaling pathway (e.g., serine/threonine kinase (AKT1/2/3), glycogen synthase kinase-3β (GSK-3β), mammalian target of rapamycin (mTOR), and p70 ribosomal protein S6 kinase (p70S6K)) are decreased in SCZ patients, suggesting that the insulin pathway is impaired in SCZ patients [39,40]. In neuronal plasma exosomes from patients with drug-naive first-episode SCZ, the ratio of phosphorylated AKT1/2/3, GSK-3β, mTOR, and p70S6K to total proteins and the ratio of phosphorylated mTOR (pS2448-mTOR) to total m-TOR are decreased [41].

## 4. Exosomes in the Diagnosis of Mood Disorders

**Figure 3:**
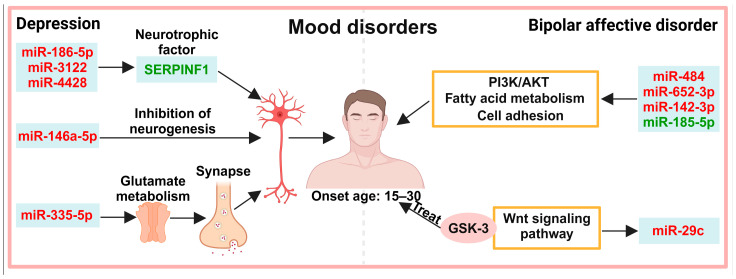
Potential exosomal diagnostic biomarkers in mood disorders. Proteins and miRNAs associated with neuroactivity, as well as miRNAs associated with PI3K/AKT, fatty acid metabolism, cell adhesion, and the Wnt signaling pathway, have been detected in patients with depression. Note: Light green boxes represent exosomal biomarkers; red font represents increased expression; green font represents decreased expression. miR: microRNA/miRNA; SERPINF1: serpin family F member 1; PI3K: phosphatidylinositide 3-kinase; AKT: serine/threonine kinase; GSK-3: glycogen synthase kinase-3.

Mood disorders include persistent excessive sadness and happiness, usually beginning between the ages of 15 and 30. Mood disorders include depression, bipolar affective disorder (BD), mania, and anxiety [42]. These disorders show a high co-occurrence rate, lack clear diagnostic criteria, and are usually associated with other serious comorbidities. To date, biomarkers for depression and BD have been investigated (Figure 3).

### 4.1. Neuronal Activity

In patients with depression, all identified exosomal biomarkers function in neurodevelopment. The serpin family F member 1 (SERPINF1)/pigment epithelium-derived factor is a kind of neuronal trophic factor, and is significantly reduced in serum exosomes from patients with major depressive disorder (MDD), whereas miR-186-5p, miR-3122, and miR-4428, which target SERPINF1, are significantly upregulated [43]. miR-146a-5p is significantly upregulated in MDD serum exosomes [44]. Overexpression of miR-146a-5p inhibits neurogenesis [45]. In MDD patients, spontaneous firing of excitatory neurons associated with cortical thickness is inhibited [44,46]. miR-335-5p targets glutamate metabotropic receptor 4 (GRM4) to regulate neuronal excitability [47], and reduced glutamate levels in specific brain regions have been implicated in the pathogenesis of depression [48,49]; miR-335-5p is significantly increased in the plasma exosomes of people with treatment-resistant depression [48]. miR-29c is activated by the Wnt signaling pathway [50,51], and in the prefrontal cortex exosomes of BD patients miR-29c is significantly increased [30].

### 4.2. Other Pathways

MiR-484, miR-652-3p, and miR-142-3p, which are associated with the phosphatidylinositide 3-kinase (PI3K) and AKT signaling pathway, fatty acid biosynthesis/metabolism pathway, and extracellular matrix and adhesion pathway, are significantly decreased in plasma exosomes from BD patients, whereas miR-185-5p is significantly increased [52].

## 5. Exosomes in the Diagnosis of Demyelinating Diseases

**Figure 4:**
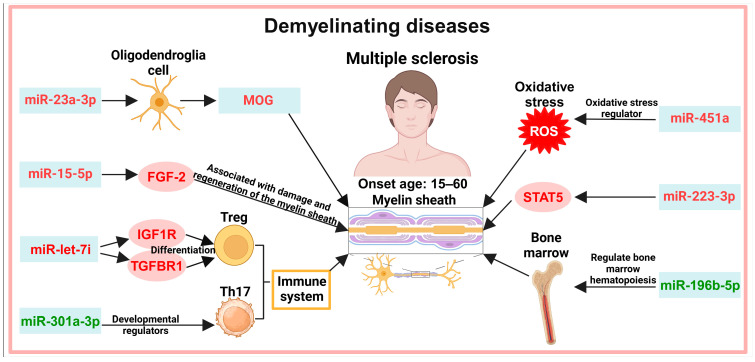
Potential exosomal diagnostic biomarkers in demyelinating diseases. MOG proteins and related miRNAs, FGF-2-related miRNAs, and immune-system-related miRNAs have been detected in exosomes of MS patients, which are directly related to myelin damage and regeneration. Abnormal expression of miRNAs related to oxidative stress and bone marrow hematopoiesis in MS patients may also be related to myelin. Note: Light green boxes represent exosomal biomarkers; red font represents increased expression; green font represents decreased expression. miR: microRNA/miRNA; MOG: myelin oligodendrocyte glycoprotein; FGF-2: fibroblast growth factor-2; IGF1R: insulin-like growth factor 1 receptor; TGFBR1: transforming growth factor β receptor 1; Treg: regulatory T cells; Th17: T cells 17; ROS: reactive oxygen species; STAT5: signal transducer and activator of transcription 5.

Demyelinating diseases, which are caused by the loss of myelin sheaths on the axonal surfaces of nerve cells, are associated with the immune system, with onset between the ages of 15 and 60. Multiple sclerosis (MS) is a T-cell mediated inflammatory autoimmune disease characterized by scattered demyelinating foci in the brain and spinal cord and damage to the CNS [53,54,55]. Exosome diagnostic biomarkers in different pathways have been identified in MS (Figure 4).

### 5.1. Myelin Demyelination

Myelin demyelination is a pathogenic mechanism in MS. Myelin is produced by oligodendrocytes, and myelin oligodendrocyte glycoprotein (MOG) is a target of cellular and humoral immune responses in MS. MS has three subtypes: relapsing–remitting multiple sclerosis (RRMS), secondary progressive multiple sclerosis (SPMS), and primary progressive multiple sclerosis (PPMS) [56,57,58]. MOG is the most immunogenic myelin phospholipid protein, and anti-MOG antibodies can lead to myelin destruction [59,60]; MOG is significantly increased in serum exosomes from patients with relapsing–remitting RRMS and patients with SPMS [61]. Fibroblast growth factor-2 (FGF-2) has been implicated in myelin destruction and regeneration, and FGF-2 in cerebrospinal fluid (CSF) can be used as a diagnostic biomarker for MS [62,63]. MiR-15-5p, which targets FGF-2 [64,65], is significantly increased in plasma exosomes from patients with multiple sclerosis or SPMS [66]. MiR-23a-3p, which is involved in the regulation of oligodendrocyte differentiation [67], is significantly increased in the cerebral white matter of MS patients [68].

### 5.2. Immune System

The balance of inflammatory T-cells and regulatory T-cells is dysregulated in MS patients, and regulatory T cells inhibit the proliferation and function of inflammatory T cells [69]. Let-7i is an miRNA and targets the insulin-like growth factor 1 receptor (IGF1R) and transforming growth factor β receptor 1 (TGFBR1), thereby inhibiting the induced differentiation of regulatory T cells (Treg). In MS plasma exosomes, let-7i is significantly increased [70]. miR-301a-3p, a developmental regulator of inflammatory CD4 helper T cells 17 (Th17) [71], is decreased in serum exosomes with RRMS [72].

### 5.3. Other Pathways

miR-196b-5p, which is associated with hematopoietic processes in the bone marrow, is decreased in serum exosomes of patients with RRMS [72]. The oxidative stress regulator miR-451a is significantly increased in RRMS plasma exosomes [66]. The signal transducer and activator of transcription 5 (STAT5) is an inflammatory regulator and transcription factor involved in the oxidative phosphorylation process associated with ROS production [73]. miR-223-3p, which targets STAT5, is increased in plasma exosomes of SPMS patients [74,75,76].

## 6. Exosomes in the Diagnosis of Neurodegenerative Diseases

**Figure 5:**
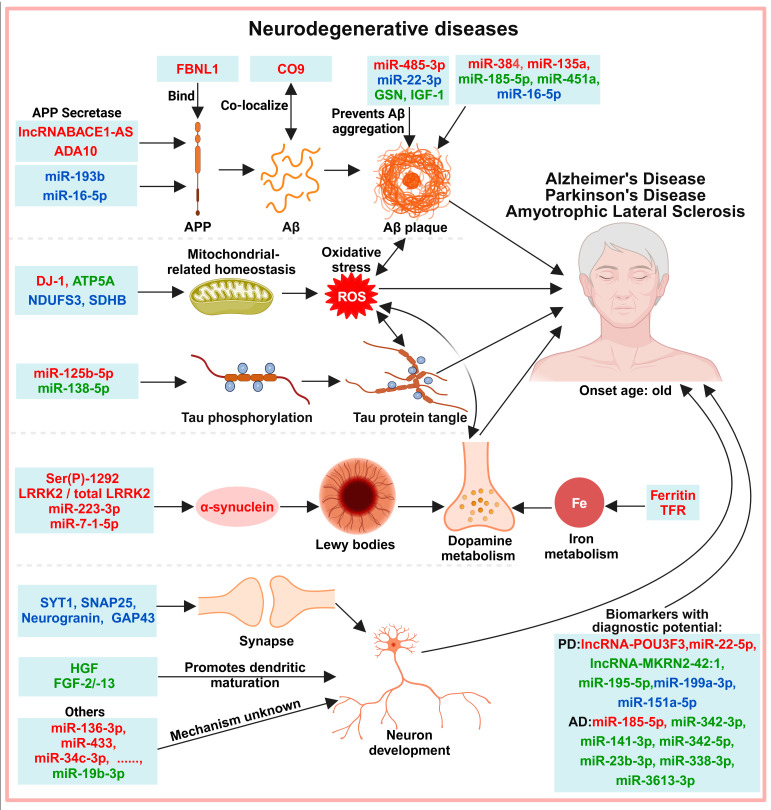
Potential exosomal diagnostic biomarkers in neurodegenerative diseases. Aβ-associated proteins and genes, Tau-associated proteins and genes, Lewy-body-associated proteins and genes, and neurodevelopmental-related proteins and genes are aberrantly expressed in exosomes from patients with neurodegenerative diseases. Note: Light green boxes represent exosomal biomarkers; red font represents increased expression; green font represents decreased expression, blue font represents both increased and decreased expression. APP: amyloid precursor protein; lncRNA BACE1-AS: (long non-coding β-secretase 1 Antisense RNA); ADA10/ADAM10: A disintegrin and metalloproteinase; FBLN1: fibulin-1; Aβ: amyloid β-protein; CO9: complement C9; GSN: gelsolin; IGF-1: insulin-like growth factor 1; DJ-1/PARK7: human protein deglycase, encoding by the PARK7 gene; ATP5A: ATP synthase F1 subunit alpha; NDUFS3: nicotinamide adenine dinucleotide (NADH) ubiquinone oxidoreductase core subunit S3; SDHB: succinate dehydrogenase complex subunit B; ROS: reactive oxygen species; Ser(P)-129 LRRK2/total LRRK2: the ratio of phosphorylated leucine-rich repeat kinase 2 (Ser(P)-129 LRRK2) to total leucine-rich repeat kinase 2 (LRRK2); TFR: ferritin receptor; SYT1: synaptotagmin 1; SNAP25: synaptosome-associated protein 25; GAP43: growth-associated protein 43; HGF: hepatocyte growth factor; FGF-2/-13: fibroblast growth factor; PD: Parkinson’s disease; AD: Alzheimer’s disease; POU3F3: POU Class 3 Homeobox 3; MKRN2: makorin ring finger protein 2.

Neurodegenerative diseases are caused by the loss of neurons and/or myelin sheaths, and the dysfunction worsens over time. The most common neurodegenerative diseases are Alzheimer’s disease (AD), Parkinson’s disease (PD), amyotrophic lateral sclerosis (ALS), dementia with Lewy bodies, frontotemporal lobe dementia, and Huntington’s disease. The major pathologic change in AD is neurofibrillary tangles caused by deposition of amyloid β-protein (Aβ) and Tau proteins [77], and clinical diagnosis is made by imaging of Aβ protein deposition with positron emission tomography (PET) and/or detection of Aβ and Tau protein concentrations in CSF [78,79]. PD is a progressive disorder characterized by resting tremor, muscle hypertonia, bradykinesia, and, eventually, gait and postural instability; the main pathogenic mechanisms of PD are neurotoxicity caused by protein misfolding and Lewy body formation [80,81], progressive lesions of dopamine (DA) neurons caused by oxidative stress [82], and impaired glutamatergic transmission in the brain [83]. ALS is caused by protein folding, oxidative stress, axonal injury, and neuroinflammation leading to degeneration of muscle motor neurons [84,85]. Pathogenesis-related and other biomarkers are identified in exosomes from neurodegenerative patients (Figure 5).

### 6.1. Protein-Misfolding-Induced Neurotoxicity

In patients with Alzheimer’s disease, Aβ (amyloid β-protein) is increased in exosomes from several sources [86,87,88,89]. Aβ is produced by hydrolysis of the transmembrane amyloid precursor protein (APP). APP is cleaved by β-secretase on the early endosome membrane; this early endosome undergoes maturation to form MVB [90]. Aβ_1–42_, the long isoform of Aβ protein, is more prone to aggregation. Aβ_1–42_ from plasma neuron exosomes can be used to differentiate pathogenic stages of AD [89]. Aβ processing and synthesis-associated proteins are also potential markers for the diagnosis of AD. A disintegrin and metalloproteinase (ADAM10) is the major α-secretase in APP processing and prevents the accumulation of Aβ in neurons [91]; gelsolin (GSN) is an Aβ-binding that prevents Aβ aggregation [92]; insulin-like growth factor 1 (IGF-1) induces the release of neuron-bound Aβ oligomers and inhibits Tau phosphorylation [93,94]. These proteins are significantly reduced in exosomes from different sources of AD patients [89,95,96]. Fibulin-1 (FBLN1), which binds to APP to regulate neuronal activity and prevent Aβ production [97,98,99], is increased in serum-derived exosomes from AD patients [100]. Complement C9 (CO9), a key subcomponent of the membrane attack complex (MAC), co-localizes with Aβ and Tau proteins in the brains of AD patients [101] and is increased in plasma exosomes [102]. Exosomal miRNAs associated with Aβ proteins are also potential diagnostic markers for AD. Plasma and salivary exosomal miR-485-3p and serum exosomal miR-22-3p, which are involved in inhibiting Aβ aggregation, are increased in AD patients [103,104,105]. Serum and plasma exosomal miR-185-5p and plasma and CSF exosomal miR-451a, which are involved in the regulation of APP hydrolysis processes, are decreased [106,107]; serum and plasma exosomal miR-384 are increased [108,109]. However, in the case of miR-193b, which targets APP, trends in CSF and serum exosomes are inconsistent [110]. Serum exosome-derived miR-135a increases in both mild cognitive impairment (MCI) [111] and AD patients [109], whereas it decreases in amnestic mild cognitive impairment (aMCI) [111]. MiR-16-5p, which targets APP, and the CSF-derived exosome miR-16-5p decrease in early-onset AD compared to healthy controls, while the difference is not significant in late-onset AD compared with healthy controls [107], and the authors suggested that it could be used to differentiate between early- and late-onset AD. β-secretase 1 (BACE1) is the secretase of APP; the expression level of lncRNA of BACE1-AS (BACE1 Antisense RNA), which is associated with Aβ processing, is increased in AD patients [112]. BACE1-AS RNA is also increased in the brain of AD patients [113,114]. The differences in the expression of the above proteins, miRNAs, and lncRNAs and their application in the diagnosis of AD need to be further investigated.

In AD patients, hyperphosphorylation of Tau leading to neurofibrillary tangles and, ultimately, neuronal apoptosis is one of the major pathological causes [77,115]. The Tau expression level in CSF is an important indicator for the diagnosis of AD patients. Exosomal Tau of neuronal origin in plasma is significantly increased in AD patients [87,88,89], and exosomal Tau proteins of metabolic origin from neuronal Tau proteins in plasma have the same diagnostic properties as Tau in CSF for AD. miR-138-5p is involved in the regulation of Tau protein phosphorylation, whereas miR-138-5p is decreased in the blood exosomes of AD patients [116].

In PD patients, aberrant protein aggregation is the main pathogenic mechanism [80]. Mutations in leucine-rich repeat kinase 2 (LRRK2) lead to abnormal protein aggregation to form Lewy bodies [117,118], and the protein ratio of urinary exosome-phosphorylated LRRK2 (Ser(P)-129 LRRK2) to total LRRK2 is significantly increased in PD patients [119]. Plasma exosomal α-synuclein is increased in PD patients compared with healthy controls [8]; serum neuronal exosomal α-synuclein is significantly higher than in the APS group [9], and serum and plasma neuronal exosomal α-synuclein are significantly lower than in the multiple system atrophy (MSA) group [8]. The expression of miR-223-3p and miR-7-1-5p, which target and regulate α-synuclein, are increased [120]. In PD patients, Tau levels in serum neuron-derived exosomes correlate with disease progression and are significantly lower in PD than in APS patients [9].

Plasma exosomal miR-16-5p and miR-22-3p levels are significantly increased in patients with medullary-onset ALS, whereas exosomal miR-16-5p is significantly lower in patients with limb-onset ALS. miR-16-5p and miR-22-3p regulate Aβ synthesis [121,122].

### 6.2. Neuronal Development and Function

Synaptotagmin 1 (SYT1), synaptosome-associated protein 25 (SNAP25), neurogranin, and growth-associated protein 43 (GAP43) are decreased in exosomes from neuronal serum of AD patients [123], whereas they are increased in CSF of AD patients [124]. Hepatocyte growth factor (HGF) and fibroblast growth factor (FGF-2/-13), which promote neurite outgrowth and dendrite maturation [125,126], are decreased in plasma exosomes from AD patients [96]. Hemoglobin (Hb) and ras suppressor protein 1 (RSU1) are increased in exosomes from various sources [102,127]. Mitochondrial dysfunction-associated nicotinamide adenine dinucleotide (NADH), ubiquinone oxidoreductase core subunit S3 (NDUFS3), and succinate dehydrogenase complex subunit B (SDHB) are decreased in plasma neuron-derived exosomes [128]. Glycoprotein Ib platelet subunit β (GP1BB), which is pathologically associated with endothelial dysfunction and cerebral amyloid angiopathy, increases in plasma exosomes [128]. PIWI-interacting RNAs (piRNAs) are associated with neurogenomic stability [129,130]. PiR-019324 decreases in cerebrospinal fluid of AD patients, and piR-019949 and piR-020364 increase in cerebrospinal fluid of AD patients [129].

In PD patients, glutamatergic neurotransmission in the brain is dysregulated [131,132]. The main pathogenic mechanism of PD is oxidative stress caused by impaired DA signaling. Both iron metabolism disorders and DJ-1 mutations contribute to oxidative stress and induce progressive lesions in nigrostriatal DA neurons [133,134]. In PD patients, ferritin and total ferritin receptor (TFR) and DJ-1 are increased in exosomes from different sources [109,135]. The oxidative stress-related proteins ATP synthase F1 subunit alpha (ATP5A), NDUFS3, and SDHB are decreased in serum exosomes [111], and miR-136-3p, miR-433, and miR-4639-5, which are associated with the dopaminergic synaptic pathway, are increased in exosomes from different sources [124,136]. In the brain of PD patients, glutamatergic neurotransmission is dysregulated [83], and the activity of the glutamatergic system is associated with PD progression [137]. Vesicular glutamate transporter-1 (VGLUT-1), an intermediate in glutamate–synaptic interactions, is significantly reduced in plasma neuron-derived exosomes from PD patients. However, excitatory amino acid transporter-2 (EAAT-2), also an intermediate in glutamate–synaptic interactions, is upregulated [137]. Acetylcholinesterase (AChE) is significantly reduced in plasma exosomes of PD patients; acetylcholinesterase is a key enzyme in biological nerve conduction that breaks down acetylcholine, terminating the excitatory effect of neurotransmitters on synaptic membranes and ensuring the normal transmission of nerve signals throughout the organism [138]. In PD patients, miR-1 and miR-19b-3p, which are associated with the neurotrophic signaling pathway, are downregulated in CSF exosomes, whereas miR-153, miR-409-3p, miR-10a-5p, and let-7g-3p are upregulated [136]; miR-128, which is specifically enriched in the brain and neurons [139,140], is upregulated in PD patients [136]. In addition, there are many miRNAs with unknown functions that also show significant changes in PD.

In ALS, miRNAs involved in the regulation of neuronal development and cell survival and proliferation have been implicated in ALS pathogenesis. miR-34a-3p, miR-199a-3p, miR-1306-3p, miR-23a-3p, and miR-146a-5p are significantly increased in plasma exosomes of ALS patients [141]. miR-34a-3p can differentiate between the diagnosis of disseminated ALS (SALS), superoxide dismutase 1 (SOD1) mutant ALS (SOD1-ALS), and complement C9 (C9) mutant ALS (C9-ALS) [141]. The plasma exosome miR-30b-5p, which functions in the axon guidance pathway, is increased in patients with ALS [141].

### 6.3. Other Molecules

The mechanism of exosomal lncRNAs on neurodegenerative diseases is unclear. lncR-POU3F3 is associated with CNS function and the lysosomal autophagy pathway [142,143]. A significant increase of lncRNA of POU Class 3 Homeobox 3 (POU3F3) and a significant decrease of lncRNA of makorin ring finger protein 2 (MKRN2)-42:1 are detected in plasma exosomes of PD patients [144]. In addition, some miRNAs with unknown functions show good diagnostic effects for neurological diseases; for example, the miRNAs in plasma exosomes miR-342-3p, miR-141-3p, miR-342-5p, miR-23b-3p, miR-338-3p, and miR-3613-3p show a high diagnostic ability of 91.9% in AD patients [145]. Serum exosomal miR-199a-3p and miR-195-5p could discriminate stage II PD from healthy populations, while miR-28-5p could discriminate stage III PD from healthy populations, and miR-22-5p and miR-151a-5p could discriminate stage IV PD from healthy populations [146].

## 7. Discussion

### 7.1. Isolation, Analysis, and Validation of Exosome Biomarkers

The diagnostic accuracy of exosomes is affected by a variety of factors, such as extraction methods, analysis, and detection methods. The main exosome extraction methods used in clinical research are ultracentrifugation and immunoaffinity- and precipitation-based extraction kits. Size exclusion chromatography, ultrafiltration, and precipitation and immunoaffinity capture can also be used in exosome extraction. Newly developed exosome extraction methods include label-free microfluidic platforms, immunoaffinity-based microfluidics, asymmetric flow field-flow fractionation, etc. Label-free microfluidic platforms separate extracellular vesicles of different sizes by an acoustic nanofilter system, which enables continuous rapid and non-contact separation of exosomes [147]. Immunoaffinity-based microfluidics combines antigen–antibody reactions and magnetic force with microfluidic chip technology to achieve specific extraction of different subpopulations of exosomes [148]. Asymmetric flow field- flow fractionation combines size, density, Brownian motion, and translational diffusion to separate extracellular vesicles down to 1 nm [149,150]. These new technologies can improve the purity and efficiency of exosome separation, and separate subpopulations of exosomes, but their role in exosome diagnosis remains to be investigated. Common detection methods for exosomes include mass spectrometry, enzyme-linked immunosorbent assay (ELISA), polymerase chain reaction (PCR), etc., as well as new quantitative analysis methods based on nanofluorescent probes, microfluidic chips, and gene chips [151,152,153,154]. Nanofluorescent probes combine with target molecules to form fluorescent complexes, and the fluorescence intensity of the complexes can be detected to quantify the target molecules [151]. A microfluidic microarray is based on the control of fluid flow and scale structure in microchannels and combines fluorescence, electrochemistry, mass spectrometry, and other methods to achieve quantitative detection of target molecules [152]. Gene microarrays and gene chips are based on the principles of complementary gene pairing, and can be used for gene quantify analysis [153,154]. These new detection methods are high throughput, high sensitivity, low sample consumption, and rapid analysis, and may play an important role in the detection of exosome-associated markers in the future.

### 7.2. Exosome Biomarkers Classification

miRNAs, genes, and proteins are three major classes of diagnostic markers in neuropsychiatric diseases. miRNA nomenclature is specific. Most genes and proteins are highly conserved, and we summarize the nomenclature of these homologue gene and protein names in different species (Table 1).

### 7.3. Ethical Concerns

When using human samples, ethical concerns should be considered. The collection and use of human exosomal samples requires strict adherence to the principles of privacy protection and informed consent. Researchers need to manage and store sample information appropriately and ensure that the participant recruitment process is fair and equitable.

## 8. Conclusions and Future Directions

Using exosomal biomarkers for assistant diagnosis not only improves diagnostic accuracy but can also be used for early diagnosis and differentiation of disease progression, which can help physicians to provide appropriate treatment plans for patients, thus supporting personalized clinical treatment strategies for patients with neuropsychiatric disorders. For example, the concentrations of neuronal-derived exosomal proteins GAP43, neurogranin, SNAP25, and SYT1 were significantly lower in AD patients than in controls, which can identify preclinical AD 5–7 years before cognitive impairment appears [123]. Serum exosomal miR-199a-3p, miR-195-5p, miR-28-5p, miR-22-5p, and miR-151a-5p can be used to distinguish different stage of PD from the healthy population [146].

Currently, there are no diagnostic markers of exosomes that have been applied to the clinical diagnosis of neuropsychiatric disorders. An important reason for this is the specificity of neurological diagnostic markers. Some markers show the same trend in different diseases; for example, in the exosomes of AD, PD, and ALS, Aβ and Tau proteins are increased [9,86,87,88,89,121], and SDHB and NDUFS3 are significantly reduced [111,128]. It is necessary to combine multiple biomarkers in the clinical diagnosis. In addition, most identified diagnostic biomarkers have only been studied in the control group and limited disease groups, and comprehensive studies of these biomarkers in different disease systems are needed.

## Figures and Tables

**Table 1:** Protein biomarkers in the diagnosis of neuropsychiatric diseases.

Symbol (Name)	Also Known as	Functions
SYT15 (Synaptotagmin 15)	SytXV, CHR10SYT, SYT15B, Sytbeta	Membrane transport proteins of the synaptotagmin family
SYT9 (Synaptotagmin 9)	SytIX, syt9a, syt9b, Syt1	Calcium ion binding activity; phospholipid binding activity; and syntaxin binding activity
STX8 (Syntaxin-8)	CARB, Hsap	Neurotransmitter release; neuronal membrane maturation
SLC18A2 (Solute carrier protein 18 A2)	PKDYS2, SVMT, VAT2, VMAT2, SVAT	Monoaminergic system
SV2C (Synaptic vesicle glycoprotein 2C)	SLC22B3, KIAA1054	Transmembrane transporter activity; protein binding
SYP (Synaptophysin)	MRX96, MRXSYP, XLID96	Synapsin, a phosphoprotein associated with synaptic vesicles
DJ-1 (Parkinsonism-associated deglycase)	DJ-1, DJ1, PARK7, GATD2, HEL-S-67p	Antioxidant protein
SERPINF1 (Serpin family F member 1)	EPC-1, OI12, OI6, PEDF, PIG35	Serine-type endopeptidase inhibitor activity; neurotrophic factors
GRM4 (Glutamate metabotropic receptor 4)	GPRC1D, MGLUR4, mGlu4	Adenylate cyclase inhibiting G protein-coupled glutamate receptor activity; G protein-coupled receptor activity
AKT (Serine/threonine kinase)	RAC, PKB, PRKBA	Protein serine/threonine/tyrosine kinase activity
GSK-3β (Glycogen synthase kinase-3β)	CiGSK; Gsk3b	p53 binding; protein serine/threonine kinase activity
MOG (Myelin oligodendrocyte glycoprotein)	BTN6, BTNL11, MOGIG2, NRCLP7	CNS-related functions, signaling receptor binding; virus receptor activity
FGF-2 (Fibroblast growth factor-2)	BFGF, FGF2, FGFB, HBGF-2	Fibroblast growth factor receptor binding; cytokine activity; integrin binding
IGF1R (Insulin-like growth factor 1 receptor)	CD221, IGFIR, IGFR, JTK13	G-protein alpha-subunit binding; protein tyrosine kinase activity
TGFBR1 (Transforming growth factor β receptor 1)	AAT5, ACVRLK4, ALK-5, ALK5, ESS1, LDS1, LDS1A, LDS2A, MSSE, SKR4, TBR-i, TBRI, TGFR-1, tbetaR-I	Protein serine/threonine kinase activity; protein serine/threonine kinase activity
STAT5 (Signal transducer and activator of transcription 5)	MGF; STAT5A, Stat92E	DNA-binding transcription factor activity, RNA polymerase II-specific
APP (Amyloid precursor protein)	AAA, ABETA, ABPP, AD1, APPI, CVAP, PN-II, PN2, alpha-sAPP, preA4	RNA polymerase II cis-regulatory region sequence-specific DNA binding; serine-type endopeptidase inhibitor activity
ADAM10 (A disintegrin and metalloproteinase)	ADA10, MADM, HsT18717, CD156C,	Endopeptidase activity; metalloendopeptidase activity; signaling receptor binding
IGF-1 (Insulin-like growth factor 1)	IGF, IGF-I, IGFI, MGF, IBP1	Insulin-like growth factor receptor binding; hormone activity
GSN (Gelsolin)	ADF, AGEL	Phosphatidylinositol 3-kinase catalytic subunit binding; calcium ion binding
FBLN1 (Fibulin-1)	FBLN, FIBL1	Extracellular matrix structural constituent; fibronectin binding; calcium ion binding
CO9 (Complement C9)	C9, ARMD15, C9D	Protein binding
BACE1 (β-secretase 1)	ASP2, BACE, HSPC104	Amyloid-beta binding; endopeptidase activity; aspartic-type endopeptidase activity
LRRK2 (Leucine-rich repeat kinase 2)	AURA17, DARDARIN, PARK8, RIPK7, ROCO2	Magnesium ion binding; actin binding
α-synuclein	SNCA, NACP, PARK1, PARK4, PD1,	Magnesium ion binding; transcription cis-regulatory region binding; protein kinase inhibitor activity
SYT1 (Synaptotagmin 1)	BAGOS, P65, SVP65, SYT, Syt1	Phosphatidylserine binding; calcium ion binding; calmodulin binding
SNAP25 (Synaptosome-associated protein 25)	CMS18, RIC-4, RIC4, SEC9, SNAP, SNAP-25, SUP, bA416n4.2, dJ1068f16.2	Voltage-gated potassium channel activity; lipid binding
GAP43 (Growth-associated protein 43)	B-50, GAP-43, PP46	Phosphatidylserine binding; calmodulin binding; lysophosphatidic acid binding
HGF (Hepatocyte growth factor)	DFNB39, F-TCF, HGFB, HPTA, SF, Tequila	Endopeptidase activity; serine-type endopeptidase activity; signaling receptor binding
FGF-13 (Fibroblast growth factor 13)	FGF2; FHF2; DEE90; FHF-2; FGF-13; XLID110; LINC00889	Microtubule binding; growth factor activity; sodium channel regulator activity; transmembrane transporter binding
RSU1 (Ras suppressor protein 1)	RSP-1, FLJ31034, Rsu-1	Protein binding
NDUFS3 (Nicotinamide adenine dinucleotide (NADH) ubiquinone oxidoreductase core subunit S3)	CI-30, MC1DN8	NADH dehydrogenase activity; oxidoreductase activity
SDHB (Succinate dehydrogenase complex subunit B)	CWS2, IP, MC2DN4, PGL4, PPGL4, SDH, SDH1, SDH2, SDHIP	Succinate dehydrogenase (quinone) activity; electron transfer activity; oxidoreductase activity
GP1BB (Glycoprotein Ib Platelet subunit β)	BDPLT1, BS, GPIBB, CD42C, GPI-b Beta	Transmembrane signaling receptor activity; identical protein binding
TFR (Total ferritin receptor)	T9; TR; TFRC; p90; CD71; TFR1; TRFR; IMD46	Virus receptor activity; double-stranded RNA binding; transferrin receptor activity
ATP5A (ATP Synthase F1 Subunit Alpha)	OMR; ORM; ATPM; MOM2; ATP5A1; hATP1; ATP5A1; MC5DN4; ATP5AL2; COXPD22; MC5DN4A; MC5DN4B; HEL-S-123m	Protease binding; protein binding; ATP binding
VGLUT-1 (Vesicular glutamate transporter-1)	SLC17A7, BNPI	Chloride channel activity; inorganic phosphate transmembrane transporter activity; L-glutamate transmembrane transporter activity; neurotransmitter transmembrane transporter activity; sodium:phosphate symporter activity
EAAT-2 (Excitatory amino acid transporter-2)	SLC1A2, GLT1; HBGT; DEE41; EAAT2; GLT-1; EIEE41	L-glutamate transmembrane transporter activity; monoatomic anion transmembrane transporter activity; neutral L-amino acid transmembrane transporter activity
AChE (Acetylcholinesterase)	ACEE, ARACHE, N-ACHE, YT	Amyloid-beta binding; acetylcholinesterase activity; cholinesterase activity; collagen binding
SOD1 (Superoxide dismutase 1)	ALS, ALS1, HEL-S-44, IPOA, SOD, STAHP, hSod1, homodimer	Superoxide dismutase activity; copper ion binding; zinc ion binding; antioxidant activity
POU3F3 (POU Class 3 Homeobox 3)	BRN1, OTF8, SNIBFIS, brain-1, oct-8	RNA polymerase II cis-regulatory region sequence-specific DNA binding; DNA-binding transcription factor activity
MKRN2 (Makorin ring finger protein 2)	HSPC070, RNF62	Metal ion binding

## Data Availability

No data were used for the research described in the article.

## Supplementary Materials

The following supporting information can be downloaded at: https://www.mdpi.com/article/10.3390/biology13060387/s1, Table S1: Exosomal diagnostic biomarker information. All exosomal biomarkers are obtained from actual patient samples, and biomarkers with significant change trends are selected for display [155,156,157,158,159,160,161,162,163,164,165,166,167,168].
