# Supplementary material for: Exosomes in the Diagnosis of Neuropsychiatric Diseases: A Review
Source: Biology (Basel). 2024 May 28;13(6):387. doi: 10.3390/biology13060387 (PMC11200774; doi:10.3390/biology13060387)
Supplement: Supplementary file 1 [file biology-13-00387-s001.zip › biology-2982199-supplementary.pdf]

Table S1 Exosomal diagnostic biomarkers information. All exosomal biomarkers are obtained from actual patient samples, and biomarkers with significant change trends are selected for display.

Targets	Functions	Tendency	AUC	No.	Source	Ref
Autism disease						
SLC18A2 (lncRNA)	Monoaminergic system	ASD>CT*	/	14	Plasma	[1]
SYT15 (lncRNA)	Membrane transport proteins of the synaptotagmin family	ASD>CT**	/			
SYT15 (mRNA)		ASD>CT**	/			
STX8 (lncRNA)	Syntaxin, neurotransmitter release, neuronal membrane maturation	ASD>CT*	/			
SV2C(lncRNA)	Synaptic vesicular glycoprotein 2C	ASD<CT**	/			
SV2C(mRNA)		ASD<CT**	/			
SYT9(lncRNA)	Membrane transport proteins of the synaptotagmin family	ASD>CT**	/			
SYT9(mRNA)		ASD>CT**	/			
SYP (lncRNA)	Synapsin, phosphoprotein associated with synaptic vesicles	ASD<CT*	/			
SYP (mRNA)		ASD<CT**	/			
mtDNA	Mitochondrial DNA	ASD>CT*	/	20	Serum	[2]
Exosome proteins (CD81, CD9)	Exosome marker proteins	ASD>CT*	/			
Schizophrenia						
DJ-1	Antioxidant protein	SCZ<CT*	/	11	Serum	[3]
miR-203a-3p	Negative regulation DJ-1	SCZ>CT*	/			
miR-497	Promote ischemic neuronal death by negatively regulating anti-apoptotic proteins, bcl-2 and bcl-w	SCZ>CT**	/	8	PFC	[4]
Total phosphorylation/total signaling protein	Insulin signal transduction	DNFES<CT*	/	24	Plasma NDESs	[5]
Phosphorylation mTOR (ps2448-mtor)/mTOR		DNFES<CT*	/			
miR-144-3p	/	SCZ>CT**	/	23	Serum	[6]
miR-206	/	SCZ>CT***	/			
miR-619-5p	/	SCZ>CT***	/			
Major depressive disorder						
SERPINF1	Neurotrophic factors	MDD<CT**	/	10	Serum	[7]
miR-186-5p	Target SERPINF1	MDD>CT*	/			
miR-3122		MDD>CT*				
miR-4428		MDD>CT*				

miR-146a-5p	Inhibitory neurogenesis	MDD>CT***	/	113	Serum	[8]
miR-335-5p	Target glutamate metabotropic receptor4 (GRM4)	TRD>CT*	/	4	Plasma	[9]
miR-1292-3p	/	TRD<CT***	/			
miR-139-5p	/	MDD>CT***	0.807	30	Serum	[10]
Bipolar disorder						
miR-29c	Induced by canonical Wnt signaling	BD>CT**	/	10	PFC	[4]
miR-185-5p	/	BD>CT***	/	69	Plasma	[11]
miR-484	/	BD<CT***	/			
miR-652-3p	/	BD<CT***	/	69	Plasma	[11]
miR-142-3p	/	BD<CT***	/			
Multiple sclerosis						
MOG	CNS related functions	Relapse RRMS>CT*	/	24	Serum	[12]
		SPMS>CT*	/	16		
miR-let-7i	Regulates MS pathogenesis by blocking the IGF1R/TGFBR1 pathway	MS>CT***	/	4	Plasma	[13]
miR-15b-5p	Target the fibroblast growth factor-2 (FGF-2)	RRMS>CT*	0.74	25	Serum	[14]
		SPMS>CT*	/			
miR-451a	Regulator of oxidative stress	RRMS>CT**	0.83			
miR-23a-3p	Oligodendrocyte differentiation, target FGF-2	SPMS>CT*	/			
miR-223-3p	Targets the transcription factor STAT5	SPMS>CT*	/			
miR-301a-3p	An endogenous regulator of Th17	Relapse RRMS<CT**	/	63	Serum	[15]
miR-196b-5p	Expresses in hematopoietic cell populations	Relapse RRMS<CT***	/			
miR-19b	/	MS>CT***	/	4	Plasma	[13]
miR-25	/	MS>CT***				
miR-92a	/	MS>CT***				
miR-122-5p	/	Relapse RRMS<CT***	/	63	Serum	[15]
		Remission RRMS<CT*				
		Relapse RRMS<Remission RRMS***				

miR-532-5p	/	Relapse RRMS<CT***	/			
		Relapse RRMS<Remission RRMS***				
miR-432-5p	/	SPMS>CT**	/	25	Serum	[14]
miR-127-3p	/	SPMS>CT**	/			
miR-370-3p	/	SPMS>CT**	/			
miR-409-3p	/	SPMS>CT**	/			
Alzheimer's disease						
oligomeric Aβ	Formed by hydrolysis of amyloid precursor protein (APP) and neurotoxic when deposited and aggregated	AD>CT*	/	5	Saliva	[16]
Aβ		AD<CT*	/			
$A\beta_{1-42}$		AD>CT****	0.96	AD: 88	Plasma NDEs	[17]
		MCI>CT**	0.71	MCI: 87		
		AD>MCI****	0.81			
$A\beta_{1-42}$		AD>CT*	/	60	Plasma	[18]
$A\beta_{1-42}$	Formed by hydrolysis of amyloid precursor protein (APP) and neurotoxic when deposited and aggregated	AD>CT***	0.93	AD: 28, aMCI: 25	Plasma NDEs	[19]
		aMCICT***	0.74			
		AD>aMCI**	0.83			
ADAM10	The α-secretase enzymes of the APP	AD<CT***	/	58	Plasma	[20]
GSN	Prevent Aβ aggregation	AD<CT**	0.70	12	Serum	[21]
IGF-1	Induce the release of Aβ oligomers bound to neurons	AD<CT**	/	24	Plasma	[22]
FBLN1	Prevent Aβ deposition in the neurons	MCI>CT**	0.81	7	Serum	[23]
CO9	Positioning with and Tau in brain	AD>CT***	/	58	Plasma	[20]
miR-485-3p	Its antisense oligonucleotide (ASO) reduced Aβ plaque accumulation, tau pathology development and neuroinflammation	AD>CT****	/	27	Saliva	[24]
		AD>CT****	/	29	Plasma	[25]
miR-22-3p	Reducing Aβ deposit	AD>CT*	/	40	Serum	[26]
miR-185-5p	Direct binding of APP 3′UTR	AD<CT*	/	3	Serum	[27]
		AD<CT**	/	35	Plasma	[28]
miR-451a	Attenuation of ADAM10 expression	AD<CT***	0.93	28	Plasma	[29]
		YOAD<CT***	0.95	30	CSF	[30]
		LOAD<CT***	0.85			
miR-384	Suppressed the mRNA	AD>CT****	0.906	45	Plasma	[31]

	And protein expression of both APP and BACE-1					
		AD>CT****	/	208	Serum	[32]
miR-193b	Repress the mRNA And protein expression of APP	MCI>CT*	/	184	Serum	[33]
		MCI>CT*	/		CSF	
		DAT>CT*	/			
		AD<CT****	/	208	Serum	[32]
miR-135a	Directly interacted with the 3′-UTR of BACE-1	MCI>CT*	/	329	CSF	[34]
		DAT>CT*	/			
		MCI>CT*	/		Serum	
		DAT>CT*	/			
		aMCI<CT*	/			
		AD>CT****	/	208	Serum	[32]
miR-16-5p	Target APP, BACE1 and MAPT transcripts	YOAD<CT*	0.76	30	CSF	[30]
		YOAD<LOAD*	/			
lncR-BACE1-AS	BACE-1 is the β-secretase of APP	AD>CT**	0.761	72	Plasma	[35]
Tau	Neurotoxic after deposition of aggregates	AD>CT***	0.89	53	Plasma NDEs	[19]
		aMCI>CT***	0.79			
		AD>aMCI**	0.72			
P-Tau		AD>CT*	/	17	Saliva	[16]
P-s396-Tau		AD>CT**	/	115	Plasma	[18]
miR-138-5p	Involved in the regulation of tau protein phosphorylation	AD<CT**	/	113	Blood	[36]
SNAP25	Synapse-associated proteins and neuregulin	AD<CT***	0.88	49	Serum NDEs	[37]
		aMCI<CT*	/			
GAP43		AD<CT***	/			
		AD<CT***	/			
Neurogranin	Synapse-associated proteins and neuregulin	AD<CT***	/	49	Serum NDEs	[37]
SYT1		AD<CT***	/			
		aMCI<CT*	/			
HGF	Promotes mature neuron extension and endrite maturation with preferential effects on hippocampal and some cortical neurons	AD<CT**	/	24	Plasma	[22]
FGF-2	Enhanced differentiation, proliferation, and	AD<CT**	/			

	survival of neuron precursors					
FGF-13		AD<CT**				
Hb	Reactive oxygen species related inflammation	AD>CT*	0.691	20	Serum NDEs	[38]
RSU1	Involved in the extracellular signal-regulated kinase (ERK) signaling pathway	AD>CT***	/	58	Plasma	[20]
NDUFS3	Mitochondria-associated proteins	AD<CT***	0.801	36	Plasma NDEs	[39]
		AD<sMCI***	/			
		AD<aMCI*	/			
SDHB		AD<CT***	0.833			
		AD<sMCI***	/			
		AD<pMCI*	/			
GP1BB	Associated with endothelial dysfunction and cerebral amyloid angiopathy pathology	AD>CT***	/	58	Plasma NDEs	[20]
piR-019324	Correlates with the stability of the neurogenome	AD<CT**	/	42	CSF	[40]
piR-019949		AD>CT***	/			
piR-020364		AD>CT***	/			
miR-342-3p	/	AD<CT***	/	113	Blood	[36]
miR-342-3p	/	AD<CT**	0.919	35	Plasma	[41]
miR-141-3p		AD<CT**				
miR-342-5p	/	AD<CT*				
miR-23b-3p	/	AD<CT*				
miR-338-3p	/	AD<CT*				
miR-3613-3p	/	AD<CT*				
miR-132-3p	/	AD<CT**	0.77	16	Plasma NDEs	[42]
miR-125b-5p	/	YOAD>CT*	0.73	30	CSF	[30]
miR-125b-5p	/	LOAD>CT*	0.78	30	CSF	[30]
miR-378a-3p	/	AD>CT***	/	40	Serum	[26]
miR-212-3p	/	AD<CT***	0.84	16	Plasma NDEs	[42]
miR-27a-3p	/	AD>CT*	/	42	CSF	[40]
miR-30a-5p	/	AD>CT*	/			
miR-34c-3p	/	AD>CT*	/			
miR-605-5p	/	YOAD<CT*	0.701	30	CSF	[30]
	/	LOAD<CT*	0.77			

miR-223	/	AD<CT**	0.875	32	Serum	[43]
miR-30b-5p	/	AD<CT**	0.880	40	Serum	[26]
miR-21-5p	/	AD<CT***	0.95	28	Plasma	[29]
miR-485-5p	/	AD>CT*	/	113	Blood	[36]
miR-29c-5p	/	AD>CT*	/			
miR-335-5p	/	AD>CT*	/			
miR-143- 3p	/	AD>CT*	/			
miR-320a	/	YOAD<CT**	0.817	28	CSF	[44]
miR-328-3p	/	YOAD<CT*	0.768			
miR-204-5p	/	YOAD<CT*	0.741			
miR-320a	/	FTD<CT*	/	12	CSF	[44]
miR-154-5p	/	Vad>CT*	/	23	Serum	[45]
Parkinson's disease						
Ser(p)-1292 LRRK2/total LRRK2	Involved in the formation of Lewy bodies	PD>CT****	0.844	16	Urine	[46]
α-synuclein		PD>CT**	0.884	80	Plasma\ serum NDEs	[47]
		PD<MSA****	0.924			
		PD>CT**	0.65	267	Plasma	[48]
		PD>CT****	/	30	Plasma	[49]
		PD>APS****	0.817	70	Serum NDEs	[50]
miR-7-1-5p	Targets the 3'UTR region of α-Synuclein mRNA	PD>CT***	/	45	Serum	[51]
miR-223-3p		PD>CT***	/			
Tau	Neurotoxic after deposition of aggregates	PD<APS****	0.856	70	Serum NDEs	[50]
DJ-1	Antioxidant protein	PD (male)>CT*	/	26	Urine	[32]
Ferritin	Related to oxidative stress	PD>CT*	0.73	43	Plasma	[52]
TFR		PD>CT*	0.812			
AChE	Cholinergic system	PD<Healthy**	/	34	Plasma	[53]
ATP5A	Mitochondria-associated proteins	PD<CT*	/	16	Serum	[34]
SDHB		PD<CT*	/			
NDUFS3		PD<CT*	/			
miR-136-3p	Dopaminergic synapse pathway	PD>CT*	/	75	CSF	[54]
miR-433		PD>CT**	/			
miR-4639-5	Reduced DJ-1 level and increased oxidative stress leading to neuronal death	PD>CT****	/	11	Plasma	[55]
EAAT-2	Glutamatergic system	PD>CT**	/	157	Plasma NDEs	[56]
VGLUT-1		PD<CT**	/			

miR-1	Neurotrophin signaling pathway	PD<CT*	0.92	75	CSF	[54]
miR-153		PD>CT**	0.78			
miR-409-3p		PD>CT**	0.97			
miR-19b-3p		PD<CT*	0.705			
miR-10a-5p		PD>CT*	0.90			
miR-let-7g-3p		PD>CT*	/			
miR-128	Involvement in cell proliferation and neurogenesis	PD<CT**	/	25	Plasma	[54]
lncR-POU3F3	/	PD>CT*	0.763	93	Plasma	[57]
lncR-MKRN2-42:1	/	PD<CT*	/	24	Plasma	[58]
miR-199a-3p	/	PD (stage2)<CT***	/	72	Serum	[59]
miR-195-5p	/	PD (stage2)<CT*	/			
miR-28-5p	/	PD (stage3)>CT****	0.746			
miR-22-5p	/	PD (stage4)>CT****	0.817			
miR-151a-5p	/	PD (stage4)<CT****	0.741			
miR-331-5p	/	PD>CT**	0.856	52	Plasma	[46]
miR-505	/	PD>CT**	0.899			
miR-331-5p	/	PD>CT**	/	209	Serum	[60]
miR-24	/	PD>CT**	/		Serum	
miR-151a-5p	/	PD>CT**	/		CSF	
miR-214	/	PD>CT**	/		Serum	
miR-485-5p	/	PD>CT**	/		CSF	
let-7d	/	PD>CT**	0.753	30	Serum	[61]
miR-22	/	PD>CT***	0.845	30	Serum	[61]
miR-23a	/	PD>CT**	0.869			
miR-24	/	PD>CT***	0.779			
miR-142-3p	/	PD>CT***	0.783			
miR-222	/	PD>CT***	0.816	72	Serum	[59]
miR-374a-5p	/	PD>CT	/			
miR-374b-5p	/	PD>CT	/			
Amyotrophic lateral sclerosis						
miR-16-5p	Target APP, BACE1	Bulbar-onset ALS>CT*	/	22	Plasma	[62, 63]
		Spinal-onset ALS<CT*				

miR-22-3p	Reducing Aβ deposit	Bulbar-onset ALS>CT*				
miR-23a-3p	Directly target ERBB4 and regulate the AKT/GSK-3β pathway	Bulbar-onset ALS>CT*				
miR-34a-3p	Correlates with cellular regulation and neural projections	SALS>SOD1-ALS*	/	81	Plasma	[64]
		SOD1-ALS<C9-ALS*				
		SOD1-ALS<CT**				
miR-199a-3p	Overexpression inhibited the cell apoptosis、promoted cell proliferation	SALS>CT***				
miR-1306-3p	Involves in the cell survival rate and inhibits the cell injury	SOD1-ALS<CT**	/			
		C9-ALS<CT**				
		SALS>SOD1-ALS**				
		SALS>C9-ALS**				
miR-30b-5p	Axon guidance pathway，ubiquitin-mediated proteolysis	SALS>CT*				
miR-146a-5p	Regulate the low molecular weight neurofilament mRNA, and immune response	ALS>CT**	/	50	Plasma	[65]
miR-4454	/	ALS<CT**	/			
miR-151a-5p	/	ALS>CT*	/			
miR-10b-5p	/	ALS<CT*	/			
miR-29b-3p	/	ALS<CT*	/			
miR-93-5p	/	Bulbar-onset ALS>CT*	/	22	Plasma	[62]

Note: *”, p<0.05; “**”, p<0.01; “***”, p<0.001; “/”, not available. "AUC" represents the Receiver Operating Characteristic (ROC) area under curve, AUC ranges from 0 to 1, closer to 1 means better prediction is obtained, "No" represents the number of patients studied in the paper; miR/miRNA: microRNA; piR/piRNA: PIWI-interacting RNA; lncR/lncRNA: long non coding RNA.

ASD: Autism Disease**;** **SYT15:** synaptotagmin 15; **SYT9:** synaptotagmin 9**;** **STX8:** syntaxin-8**;** **SLC18A2:** solute carrier protein 18 A2; **SV2C:** synaptic vesicle glycoprotein 2C**;** **mtDNA:** mitochondrial DNA**;** **SCZ:** Schizophrenia**;** **DNFES:** drug-naive first episode Schizophrenia **;** **PFC:** prefrontal cortex**;** **NDEs:** neuronal-derived exosomes**;** **MDD:** major depressive disorder; **SERPINF1:** serpin family F member 1**;** **TRD:** treatment-resistant depression**;** **NDEs,** neuron-derived exosomes; **FTD:** frontotemporal dementia; **VaD:** vascular dementiame; **BD:** Bipolar disorder**;** **MOG:** myelin oligodendrocyte glycoprotein**;** **CNS:** central nervous system**;** **RRMS:** relapsing remitting Multiple Sclerosis**;** **SPMS:** secondary progressive Multiple Sclerosis**;** **MS:** Multiple sclerosis**;** **FGF:** fibro blast growth factor**;** **STAT5:** signal transducer and activator of transcription 5**;** **AD:** Alzheimer's Disease **;** **Aβ:** amyloid β-protein**;** **APP:** amyloid precursor protein**;** **MCI:** mild cognitive impairment**;** **ADAM10:** aisintegrin and metalloproteinase domain 10**;** **GSN:** gelsolin; **IGF-1:** type 1 insulin-like growth factor**;** **FBLN1:** fibulin 1**;** **CO9/C9:** complement component C9**;** **ASO:** antisense oligonucleotide**;** **UTR:** Untranslated Region**;** **YOAD:** young-onset Alzheimer's Disease**;** **LOAD:** late-onset Alzheimer's Disease**;** **CSF:** cerebrospinal fluid**;** **BACE1:** β-secretase 1; **DAT:** dementia of the Alzheimer type**;** **MAPT:** microtubule-associated protein tau; **P-Tau:** phosphorylation tau protein; **aMCI:** amnestic mild cognitive impairment **;** **SNAP25:** synaptosome associated protein 25**;** **GAP43:** growth associated protein 43**;** **SYT1:** synaptotagmin 1**;** **HGF:** hepatocyte growth factor**;** **FGF:** fibroblast growth factor**;** **Hb:** hemoglobin**;** **RSU1:** ras suppressor protein 1**;** **NDUFS3:** NADH ubiquinone oxidoreductase core subunit S3**;** **SDHB:** succinate

dehydrogenase domplex subunit B**;** **ERK:** extracellular regulated protein kinases**;** **sMCI:** stable Mild Cognitive Impairment**;** **pMCI:** progressive Mild Cognitive Impairment**;** **GP1BB: glycoprotein ib platelet subunit β; FTD:** Frontotemporal Dementia**;** **VaD:** Vascular Dementia**;** **PD:** Parkinson's Disease**;** **LRRK2:** leucine-rich repeat kinase 2**;** **MSA:** Multiple System Atroph**;** **APS:** Atypical Parkinsonian Syndromes**;** **TFR:** transferrin receptor**;** **AChE:** acetylcholinesterase; **EAAT-2:** excitatory amino acid transporter-2**;** **VGLUT-1:** glutamate transporter-1**;** **POU3F3:** POU class 3 homeobox 3; **MKRN2:** makorin ring finger protein 2; **ALS:** Amyotrophic Lateral Sclerosis**;** **AKT:** Serine/threonine kinase; GSK-3β**:** glycogen synthase kinase-3β**;** **SOD1:** Superoxide dismutase 1**:** **SALS:** sporadic Amyotrophic Lateral Sclerosis**.**

References

1. Fang, Y.; Wan, C.; Wen, Y.; Wu, Z.; Pan, J.; Zhong, M.; Zhong, N., Autism-associated synaptic vesicle transcripts are differentially expressed in maternal plasma exosomes of physiopathologic pregnancies. *J Transl Med* **2021,** *19* (1), 154.
2. Tsilioni, I.; Theoharides, T. C., Extracellular vesicles are increased in the serum of children with autism spectrum disorder, contain mitochondrial DNA, and stimulate human microglia to secrete IL-1β. *J Neuroinflammation* **2018,** *15* (1), 239.
3. Tsoporis, J. N.; Ektesabi, A. M.; Gupta, S.; Izhar, S.; Salpeas, V.; Rizos, I. K.; Kympouropoulos, S. P.; Dos Santos, C. C.; Parker, T. G.; Rizos, E., A longitudinal study of alterations of circulating DJ-1 and miR-203a-3p in association to olanzapine medication in a sample of first episode patients with schizophrenia. *J Psychiatr Res* **2022,** *146*, 109-117.
4. Banigan, M. G.; Kao, P. F.; Kozubek, J. A.; Winslow, A. R.; Medina, J.; Costa, J.; Schmitt, A.; Schneider, A.; Cabral, H.; Cagsal-Getkin, O.; et al. Differential expression of exosomal microRNAs in prefrontal cortices of schizophrenia and bipolar disorder patients. *PLoS One* **2013,** *8* (1), e48814.
5. Kapogiannis, D.; Dobrowolny, H.; Tran, J.; Mustapic, M.; Frodl, T.; Meyer-Lotz, G.; Schiltz, K.; Schanze, D.; Rietschel, M.; Bernstein, H. G.; Steiner, J., Insulin-signaling abnormalities in drug-naive first-episode schizophrenia: transduction protein analyses in extracellular vesicles of putative neuronal origin. *Eur Psychiatry* **2019,** *62*, 124-129.
6. Du, Y.; Yu, Y.; Hu, Y.; Li, X. W.; Wei, Z. X.; Pan, R. Y.; Li, X. S.; Zheng, G.-E.; Qin, X. Y.; Liu, Q.-S.; Cheng, Y., Genome-wide, integrative analysis implicates exosome-derived microRNA dysregulation in schizophrenia. *Schizophr Bull* **2019**, *45* (6), 1257-1266.
7. Jiang, M.; Gu, Y. F.; Cai, J. F.; Wang, A.; He, Y.; Feng, Y. L., MiR-186-5p dysregulation leads to depression-like behavior by de-repressing SERPINF1 in hippocampus. *Neuroscience* **2021**, *479*, 48-59.
8. Deng, Y.; Gong, P.; Han, S.; Zhang, J.; Zhang, S.; Zhang, B.; Lin, Y.; Xu, K.; Wen, G.; Liu, K., Reduced cerebral cortex thickness is related to overexpression of exosomal miR-146a-5p in medication-free patients with major depressive disorder. *Psychol Med* **2022**, *53* (13), 6253-6260.
9. Li, L.D.; Naveed, M.; Du, Z. W.; Ding, H.; Gu, K.; Wei, L. L.; Zhou, Y. P.; Meng, F.; Wang, C.; Han, F.; Zhou, Q. G.; Zhang, J., Abnormal expression profile of plasma-derived exosomal microRNAs in patients with treatment-resistant depression. *Hum Genomics* **2021**, *15* (1), 55.
10. Liang, J. Q.; Liao, H. R.; Xu, C. X.; Li, X. L.; Wei, Z. X.; Xie, G. J.; Cheng, Y., Serum exosome-derived miR-139-5p as a potential biomarker for major depressive disorder. Neuropsychiatric *Neuropsychiatr Dis Treat* **2020**, *16*, 2689-2693.
11. Ceylan, D.; Tufekci, K. U.; Keskinoglu, P.; Genc, S.; Özerdem, A., Circulating exosomal microRNAs in bipolar disorder. *J Affect Disord* **2020**, *262*, 99-107.
12. Galazka, G.; Mycko, M. P.; Selmaj, I.; Raine, C. S.; Selmaj, K. W., Multiple sclerosis: serum-derived exosomes express myelin proteins. *Mult Scler* **2018**, *24* (4), 449-458.
13. Kimura, K.; Hohjoh, H.; Fukuoka, M.; Sato, W.; Oki, S.; Tomi, C.; Yamaguchi, H.; Kondo, T.; Takahashi, R.; Yamamura, T., Circulating exosomes suppress the induction of regulatory T cells via let-7i in multiple sclerosis. *Nat Commun* **2018**, *9* (1), 17.
14. Ebrahimkhani, S.; Vafaee, F.; Young, P. E.; Hur, S. S. J.; Hawke, S.; Devenney, E.; Beadnall, H.; Barnett, M. H.; Suter, C. M.; Buckland, M. E., Exosomal microRNA signatures in multiple sclerosis reflect disease status. *Sci Rep* **2017**, *7* (1), 14293.
15. Selmaj, I.; Cichalewska, M.; Namiecinska, M.; Galazka, G.; Horzelski, W.; Selmaj, K. W.; Mycko, M. P., Global exosome transcriptome profiling reveals biomarkers for multiple sclerosis. *Ann Neuro* **2017**, *81* (5), 703-717.

16. Rani, K.; Rastogi, S.; Vishwakarma, P.; Bharti, P. S.; Sharma, V.; Renu, K.; Modi, G. P.; Vishnu, V. Y.; Chatterjee, P.; Dey, A. B.; et al. A novel approach to correlate the salivary exosomes and their protein cargo in the progression of cognitive impairment into Alzheimer's disease. *J Neurosci Methods* **2021**, *347*, 108980.
17. Zhao, A.; Li, Y.; Yan, Y.; Qiu, Y.; Li, B.; Xu, W.; Wang, Y.; Liu, J.; Deng, Y., Increased prediction value of biomarker combinations for the conversion of mild cognitive impairment to Alzheimer's dementia. *Transl Neurodegener* **2020,** *9* (1), 30.
18. Sun, R.; Wang, H.; Shi, Y.; Sun, Z.; Jiang, H.; Zhang, J., Changes in the morphology, number, and pathological protein levels of plasma exosomes may help diagnose Alzheimer's disease. *J Alzheimers Dis* **2020,** *73* (3), 909-917.
19. Jia, L.; Qiu, Q.; Zhang, H.; Chu, L.; Du, Y.; Zhang, J.; Zhou, C.; Liang, F.; Shi, S.; Wang, S.; et al. Concordance between the assessment of $A\beta_{42}$, T-tau, and P-T181-tau in peripheral blood neuronal-derived exosomes and cerebrospinal fluid. *Alzheimers Dement* **2019,** *15* (8), 1071-1080.
20. Cai, H.; Pang, Y.; Wang, Q.; Qin, W.; Wei, C.; Li, Y.; Li, T.; Li, F.; Wang, Q.; Li, Y.; Wei, Y.; Jia, L., Proteomic profiling of circulating plasma exosomes reveals novel biomarkers of Alzheimer's disease. *Alzheimers Res Ther* **2022,** *14* (1), 181.
21. Soares Martins, T.; Marcalo, R.; Ferreira, M.; Vaz, M.; Silva, R. M.; Martins Rosa, I.; Vogelgsang, J.; Wiltfang, J.; Cruz e Silva, O. A. B.; Henriques, A. G., Exosomal Aβ-binding proteins identified by "In Silico" analysis represent putative blood-derived biomarker candidates for Alzheimer´s disease. *Int J Mol Sci* **2021,** *22* (8), 3933.
22. Goetzl, E. J.; Nogueras-Ortiz, C.; Mustapic, M.; Mullins, R. J.; Abner, E. L.; Schwartz, J. B.; Kapogiannis, D., Deficient neurotrophic factors of CSPG4 - type neural cell exosomes in Alzheimer disease. *FASEB J* **2018,** *33* (1), 231-238.
23. Lai, Y.-J.; Chen, B.; Song, L.; Yang, J.; Zhou, W.-Y.; Cheng, Y.-Y., Proteomics of serum exosomes identified fibulin-1 as a novel biomarker for mild cognitive impairment. *Neural Regen Res* **2023,** *18* (3), 587-593.
24. Ryu, I. S.; Kim, D. H.; Ro, J.-Y.; Park, B.-G.; Kim, S. H.; Im, J.-Y.; Lee, J.-Y.; Yoon, S. J.; Kang, H.; Iwatsubo, T.; et al. The microRNA-485-3p concentration in salivary exosome-enriched extracellular vesicles is related to amyloid β deposition in the brain of patients with Alzheimer's disease. *Clin Biochem* **2023,** *118*, 110603.
25. Koh, H.; Lee, S.; Lee, H.; Min, J.-W.; Iwatsubo, T.; Teunissen, C.; Cho, H.-J.; Ryu, J.-H., Targeting microRNA-485-3p blocks Alzheimer's disease progression. *Int J Mol Sci* **2021,** *22* (23), 13136.
26. Dong, Z.; Gu, H.; Guo, Q.; Liang, S.; Xue, J.; Yao, F.; Liu, X.; Li, F.; Liu, H.; Sun, L.; Zhao, K., Profiling of serum exosome miRNA reveals the potential of a miRNA panel as diagnostic biomarker for Alzheimer's disease. *Mol Neurobiol* **2021,** *58* (7), 3084-3094.
27. Leng, B.; Sun, H.; Li, M.; Zhao, J.; Liu, X.; Yao, R.; Shen, T.; Li, Z.; Zhang, J., Blood neuro exosomal excitatory amino acid transporter-2 is associated with cognitive decline in Parkinson's disease with RBD. *Front Aging Neurosci* **2022**, *14*, 952368.
28. Squadrito, M. L.; Baer, C.; Burdet, F.; Maderna, C.; Gilfillan, G. D.; Lyle, R.; Ibberson, M.; De Palma, M., Endogenous RNAs modulate microRNA sorting to exosomes and transfer to acceptor cells. *Cell Rep* **2014**, *8* (5), 1432-46.
29. Gámez Valero, A.; Campdelacreu, J.; Vilas, D.; Ispierto, L.; Rene, R.; Alvarez, R.; Armengol, M. P.; Borràs, F. E.; Beyer, K., Exploratory study on microRNA profiles from plasma-derived extracellular vesicles in Alzheimer's disease and dementia with Lewy bodies. *Transl Neurodegener* **2019**, *3* (8), 31.
30. McKeever, P. M.; Schneider, R.; Taghdiri, F.; Weichert, A.; Multani, N.; Brown, R. A.; Boxer, A. L.; Karydas, A.; Miller, B.; Robertson, J.; Tartaglia, M. C., MicroRNA expression levels are altered in the cerebrospinal fluid of patients with young-onset Alzheimer's disease. *Mol Neurobiol* **2018**, *55* (12), 8826-8841.

31. Li, Y.; Meng, S.; Di, W.; Xia, M.; Dong, L.; Zhao, Y.; Ling, S.; He, J.; Xue, X.; Chen, X.; Liu, C., Amyloid-β protein and microRNA-384 in NCAM-Labeled exosomes from peripheral blood are potential diagnostic markers for Alzheimer's disease. *CNS Neurosci Ther* **2022**, *28* (7), 1093-1107.
32. Ho, D. H.; Yi, S.; Seo, H.; Son, I.; Seol, W., Increased DJ-1 in urine exosome of Korean males with Parkinson's disease. *Biomed Res Int* **2014**, 2014, 704678.
33. Liu, C. G.; Zhao, Y.; Lu, Y.; Wang, P. C.; Cao, D. Y., ABCA1-labeled exosomes in serum contain higher microRNA-193b levels in Alzheimer's disease. *Biomed Res Int* **2021**, *2021*, 1-10.
34. Picca, A.; Guerra, F.; Calvani, R.; Marini, F.; Biancolillo, A.; Landi, G.; Beli, R.; Landi, F.; BeRNAbei, R.; Bentivoglio, A. R., et al. Mitochondrial signatures in circulating extracellular vesicles of older adults with Parkinson's disease: results from the exosomes in Parkinson's disease study. *J Clini Med* **2020**, *9* (2), 504.
35. Wang, D.; Wang, P.; Bian, X.; Xu, S.; Zhou, Q.; Zhang, Y.; Ding, M.; Han, M.; Huang, L.; Bi, J.; Jia, Y.; Xie, Z., Elevated plasma levels of exosomal BACE1-AS combined with the volume and thickness of the right entorhinal cortex may serve as a biomarker for the detection of Alzheimer's disease. *Mol Med Rep* **2020**, *22* (1), 227-238.
36. Jia, L.; Zhu, M.; Yang, J.; Pang, Y.; Wang, Q.; Li, T.; Li, F.; Wang, Q.; Li, Y.; Wei, Y., Exosomal microRNA-based predictive model for preclinical Alzheimer's disease: a multicenter study. *Biol Psychiatry* **2022,** *92* (1), 44-53.
37. Reddy, P. H.; Mani, G.; Park, B. S.; Jacques, J.; Murdoch, G.; Whetsell, W., Jr.; Kaye, J.; Manczak, M., Differential loss of synaptic proteins in Alzheimer's disease: implications for synaptic dysfunction. *J Alzheimers Dis* **2005**, *7* (2), 103-17; 173-80.
38. Arioz, B. I.; Tufekci, K. U.; Olcum, M.; Durur, D. Y.; Akarlar, B. A.; Ozlu, N.; Bagriyanik, H. A.; Keskinoglu, P.; Yener, G.; Genc, S., Proteome profiling of neuron-derived exosomes in Alzheimer's disease reveals hemoglobin as a potential biomarker. *Neurosci Lett* **2021**, *755*, 135914.
39. Chi, H.; Yao, R.; Sun, C.; Leng, B.; Shen, T.; Wang, T.; Zhang, S.; Li, M.; Yang, Y.; Sun, H.; Li, Z.; Zhang, J., Blood neuroexosomal mitochondrial proteins predict Alzheimer disease in diabetes. *Diabetes* **2022**, *71* (6), 1313-1323.
40. Jain, G.; Stuendl, A.; Rao, P.; Berulava, T.; Pena Centeno, T.; Kaurani, L.; Burkhardt, S.; Delalle, I.; Kornhuber, J.; Hüll, M.; Maier, W.; et al. A combined miRNA-piRNA signature to detect Alzheimer's disease. *Transl Psychiatry* **2019**, *9* (1), 250.
41. He, S.; Huang, L.; Shao, C.; Nie, T.; Xia, L.; Cui, B.; Lu, F.; Zhu, L.; Chen, B.; Yang, Q., Several miRNAs derived from serum extracellular vesicles are potential biomarkers for early diagnosis and progression of Parkinson's disease. *Transl Neurodegener* **2021**, *10* (1), 25.
42. Cha, D. J.; Mengel, D.; Mustapic, M.; Liu, W.; Selkoe, D. J.; Kapogiannis, D.; Galasko, D.; Rissman, R. A.; Bennett, D. A.; Walsh, D. M., miR-212 and miR-132 are downregulated in neurally derived plasma exosomes of Alzheimer's patients. *Front Neurosci* **2019,** *13*, 1208.
43. Wei, H.; Xu, Y.; Xu, W.; Zhou, Q.; Chen, Q.; Yang, M.; Feng, F.; Liu, Y.; Zhu, X.; Yu, M.; Li, Y., Serum exosomal miR-223 serves as a potential diagnostic and prognostic biomarker for dementia. *Neuroscience* **2018**, *379*, 167-176.
44. Tan, Y. J.; Wong, B.; Y. X.; Vaidyanathan, R.; Sreejith, S.; Chia, S. Y.; Kandiah, N.; Ng, A. S. L.; Zeng, L., Altered cerebrospinal fluid exosomal microRNA levels in young-onset Alzheimer's disease and frontotemporal dementia. *J Alzheimer's Dis Rep* **2021**, *5* (1), 805-813.
45. Han, X.; Zhou, L.; Tu, Y.; Wei, J.; Zhang, J.; Jiang, G.; Shi, Q.; Ying, H., Circulating exo-miR-154-5p regulates vascular dementia through endothelial progenitor cell-mediated angiogenesis. *Front Cell Neurosci* **2022**, *16*, 881175.
46. Yao, Y. F.; Qu, M. W.; Li, G. C.; Zhang, F. B.; Rui, H. C., Circulating exosomal miRNAs as diagnostic biomarkers in Parkinson's disease. *Eur Rev Med Pharmacol Sci* **2018**, *22* (16), 5278-5283.
47. Dutta, S.; Hornung, S.; Kruayatidee, A.; Maina, K. N.; Rosario, I.; Paul, K. C.; Wong, D. Y.; Duarte Folle, A.; Markovic, D.; Palma, J. A.; et al. α-synuclein in blood exosomes immunoprecipitated using neuronal and

oligodendroglial markers distinguishes Parkinson's disease from multiple system atrophy. *Acta Neuropathol* **2021**, *142* (3), 495-511.

48. Shi, M.; Liu, C.; Cook, T. J.; Bullock, K. M.; Zhao, Y.; Ginghina, C.; Li, Y.; Aro, P.; Dator, R.; He, C.; et al. Plasma exosomal α-synuclein is likely CNS-derived and increased in Parkinson's disease. *Acta Neuropathol* **2014**, *128* (5), 639-650.
49. Kluge, A.; Bunk, J.; Schaeffer, E.; Drobny, A.; Xiang, W.; Knacke, H.; Bub, S.; Lückstädt, W.; Arnold, P.; Lucius, R.; et al. Detection of neuron-derived pathological α-synuclein in blood. *Brain* **2022,** *145* (9), 3058-3071.
50. Meloni, M.; Agliardi, C.; Guerini, F. R.; Zanzottera, M.; Bolognesi, E.; Picciolini, S.; Marano, M.; Magliozzi, A.; DiFonzo, A.; Arighi, A.; et al. α-synuclein and tau aggregates in NDEVs differentiate Parkinson's disease from atypical parkinsonisms. *Neurobiol Dis* **2023**, *176*, 105947.
51. Citterio, L. A.; Mancuso, R.; Agostini, S.; Meloni, M.; Clerici, M., Serum and exosomal miR-7-1-5p and miR-223-3p as possible biomarkers for Parkinson's disease. *Biomolecules* **2023**, *13* (5), 865.
52. Chen, Z. T.; Pan, C. Z.; Ruan, X. L.; Lei, L. P.; Lin, S. M.; Wang, Y. Z.; Zhao, Z. H., Evaluation of ferritin and TFR level in plasma neural-derived exosomes as potential markers of Parkinson's disease. *Front Aging Neurosci* **2023**, *15*, 1216905.
53. Jeong, S.; Shim, K. H.; Kim, D.; Bae, H.; Jeong, D. E.; Kang, M. J.; An, S. S. A., Assessment of acetylcholinesterase activity in CD9-positive exosomes from patients with Parkinson's disease. *Front Aging neurosci* 2024, 16, 1332455.
54. Bhattacharyya, P.; Biswas, A.; Biswas, S. C., Brain-enriched miR-128: Reduced in exosomes from Parkinson's patient plasma, improves synaptic integrity, and prevents 6-OHDA mediated neuronal apoptosis. *Front Cell Neurosci* **2023**, *16*, 1037903.
55. He, L.; Chen, Y.; Lin, S.; Shen, R.; Pan, H.; Zhou, Y.; Wang, Y.; Chen, S.; Ding, J., Regulation of has-miR-4639-5p expression and its potential role in the pathogenesis of Parkinson's disease. *Aging Cell* **2023**, *22* (6), e13840.
56. Shim, K. H.; Go, H. G.; Bae, H.; Jeong, D. E.; Kim, D.; Youn, Y. C. Kim, S.; An, S. S. A.; Kang, M. J., Decreased exosomal acetylcholinesterase activity in the plasma of patients With Parkinson's disease. *Front Aging Neurosci* **2021**, *28* (13), 665400.
57. Shan, T. D.; Xu, J. H.; Yu, T.; Li, J. Y.; Zhao, L. N.; Ouyang, H.; Luo, S.; Lu, X. J.; Huang, C. Z.; Lan, Q. S.; et al. Knockdown of linc-POU3F3 suppresses the proliferation, apoptosis, and migration resistance of colorectal cancer. *Oncotarget* **2016**, *7* (1), 961-75.
58. Wang, Q.; Han, C. L.; Wang, K. L.; Sui, Y. P.; Li, Z. B.; Chen, N.; Fan, S. Y.; Shimabukuro, M.; Wang, F.; Meng, F. G., Integrated analysis of exosomal lncRNA and mRNA expression profiles reveals the involvement of lnc-MKRN2-42:1 in the pathogenesis of Parkinson's disease. *CNS Neurosci Ther* 2020, 26 (5), 527-537.
59. He, S.; Huang, L.; Shao, C.; Nie, T.; Xia, L.; Cui, B.; Lu, F.; Zhu, L.; Chen, B.; Yang, Q., Several miRNAs derived from serum extracellular vesicles are potential biomarkers for early diagnosis and progression of Parkinson's disease. *Transl Neurodegener* 2021, 10 (1), 25.
60. Wang, L.; Zhang, L., Circulating Exosomal miRNA as Diagnostic Biomarkers of Neurodegenerative Diseases. *Front Mol Neurosci* **2020**, *13*, 53.
61. Tong, G.; Zhang, P.; Hu, W.; Zhang, K.; Chen, X.; Aasly, J., Diagnostic test to identify Parkinson's disease from the blood sera of chinese population: a cross-sectional study. *Parkinson's Dis* **2022**, *2022*, 1-8.
62. Liu, Y.; Ding, M.; Pan, S.; Zhou, R.; Yao, J.; Fu, R.; Yu, H.; Lu, Z., MicroRNA-23a-3p is upregulated in plasma exosomes of bulbar-onset ALS patients and targets ERBB4. *Neuroscience* **2023**, *524*, 65-78.
63. Parsi, S.; Smith, P. Y.; Goupil, C.; Dorval, V.; Hébert, S. S., Preclinical evaluation of miR-15/107 family members as multifactorial drug targets for Alzheimer's disease. *Mol Ther Nucleic acids* **2015**, *4* (10), e256.

64. Zou, J.; Guo, Y.; Wei, L.; Yu, F.; Yu, B.; Xu, A., Long noncoding RNA POU3F3 and α-Synuclein in plasma L1CAM exosomes combined with β-glucocerebrosidase activity: potential predictors of Parkinson ’ s disease. *Neurotherapeutics* **2020**, *17* (3), 1104-1119.
65. Barbagallo, C.; Mostile, G.; Baglieri, G.; Giunta, F.; Luca, A.; Raciti, L.; Zappia, M.; Purrello, M.; Ragusa, M.; Nicoletti, A., Specific signatures of serum miRNAs as potential biomarkers to discriminate clinically similar neurodegenerative and vascular-related diseases. *Cell Mol Neurobiol* **2019**, *40* (4), 531-546.
